# Expression of Yeast NDI1 Rescues a *Drosophila* Complex I Assembly Defect

**DOI:** 10.1371/journal.pone.0050644

**Published:** 2012-11-30

**Authors:** Jaehyoung Cho, Jae H. Hur, Jacqueline Graniel, Seymour Benzer, David W. Walker

**Affiliations:** 1 Department of Integrative Biology and Physiology, University of California Los Angeles, Los Angeles, California, United States of America; 2 Division of Biology, California Institute of Technology, Pasadena, California, United States of America; 3 Molecular Biology Institute, University of California Los Angeles, Los Angeles, California, United States of America; Ben-Gurion University of the Negev, Israel

## Abstract

Defects in mitochondrial electron transport chain (ETC) function have been implicated in a number of neurodegenerative disorders, cancer, and aging. Mitochondrial complex I (NADH dehydrogenase) is the largest and most complicated enzyme of the ETC with 45 subunits originating from two separate genomes. The biogenesis of complex I is an intricate process that requires multiple steps, subassemblies, and assembly factors. Here, we report the generation and characterization of a *Drosophila* model of complex I assembly factor deficiency. We show that *CG7598* (*dCIA30*), the *Drosophila* homolog of human complex I assembly factor *Ndufaf1*, is necessary for proper complex I assembly. Reduced expression of *dCIA30* results in the loss of the complex I holoenzyme band in blue-native polyacrylamide gel electrophoresis and loss of NADH:ubiquinone oxidoreductase activity in isolated mitochondria. The complex I assembly defect, caused by mutation or RNAi of *dCIA30,* has repercussions both during development and adulthood in *Drosophila*, including developmental arrest at the pupal stage and reduced stress resistance during adulthood. Expression of the single-subunit yeast alternative NADH dehydrogenase, *Ndi1*, can partially or wholly rescue phenotypes associated with the complex I assembly defect. Our work shows that *CG7598/dCIA30* is a functional homolog of *Ndufaf1* and adds to the accumulating evidence that transgenic NDI1 expression is a viable therapy for disorders arising from complex I deficiency.

## Introduction

Alterations in mitochondrial energy metabolism have been implicated in aging and age-onset disease [1]. Mitochondrial complex I (NADH:ubiquinone oxidoreductase/NADH dehydrogenase), is one of the most complicated enzymes of the eukaryotic cell, with more than 40 subunits originating from two separate genomes [2] and a still growing list of accessory and assembly factors [3]. Functioning as the major entry site of electrons from NADH into the mitochondrial electron transport chain (ETC), the fully assembled enzyme is thought to be L-shaped, embedded in the mitochondrial inner membrane by a hydrophobic membrane arm with a hydrophilic peripheral arm protruding perpendicularly into the mitochondrial matrix [4]. A current model of biogenesis of human complex I supports the assembly of the membrane arm in a stepwise process with subsequent addition of a partially, independently assembled peripheral arm and additional nuclear encoded subunits to complete the holoenzyme [5], [6]. Moreover, recent studies have shown that complex I subassemblies can act as scaffolds for the assembly of respiratory supercomplexes consisting of multiple ETC complexes [7].

Proper assembly of complex I is intimately associated with the presence of a multitude of assembly factors and chaperones, and their loss results in diseases that mimic complex I subunit defects [3]. Complex I assembly factors were first isolated in studies of *Neurospora crassa* mutants that accumulated complex I subassemblies, with associated assembly factors, due to a mutation in a membrane arm subunit [8]. Of the two complex I intermediate associated (CIA) proteins identified in this study, only the 30 kDa protein (CIA30) has been shown to have a human homolog (NDUFAF1) [9] that functions as an assembly factor, interacting with mid-stage membrane arm subassemblies but not with a fully assembled holoenzyme or a late-stage subunit. A patient with mutations in *Ndufaf1* has also been described who shows severely reduced levels of complex I holoenzyme and suffers from cardioencephalomyopathy [10]. Complete deletion of *CIA30* in *N. crassa* result in complete loss of complex I and respiration exclusively via an alternative internal NADH:ubiquinone oxidoreductase [8].

Unlike mitochondrial complex I, flavone-sensitive, rotenone-insensitive, single-subunit, alternative internal NADH dehydrogenase (*Ndi1*) genes are restricted to plant and fungal mitochondria where they function as NADH dehydrogenases without translocating protons across the inner mitochondrial membrane [11]–[13]. In fungus, NDI1 activity has been shown to be sufficient to complement complete loss of complex I holoenzyme [14], and some yeast, such as *Saccharomyces cerevisiae* lack a multi-subunit mitochondrial complex I entirely [15]. Due to its much simpler genetics and functionality in non-native systems [16], transgenic NDI1 has shown to be highly effective as a therapeutic tool for complex I associated diseases in non-fungal systems, including nematodes [17], arthropods [18], and mammals [19]–[23]. Moreover, the contrasting sensitivity to inhibitors between NDI1 (flavone sensitive, rotenone insensitive) and endogenous complex I (rotenone sensitive, flavone insensitive) allows for accurate assessment of the contribution of NADH:ubiquinone oxidoreductase activity from the two sources.

Previous work from our lab and others has shown that expression of *Ndi1* in the fruit fly, *Drosophila melanogaster*, can increase metabolic activity and lifespan [24], [25]. Here, we have expanded upon this paradigm by examining the impact of exogenous *Ndi1* expression in flies with a complex I assembly defect. To do so, we first characterized the consequences of reduced expression of the *Drosophila* homolog of the complex I assembly factor *Ndufaf1*/*CIA30*, *CG7598* (*dCIA30*). We demonstrate that flies carrying a mutation in *dCIA30* display a reduction of complex I holoenzyme in blue native polyacrylamide gel electrophoresis (BN-PAGE) to undetectable levels. Moreover, mitochondria isolated from larvae with *dCIA30* knock down show drastic reductions in endogenous rotenone sensitive, flavone insensitive NADH:ubiquinone oxidoreductase activity. In addition, RNAi knock down of *dCIA30* allows for development into adulthood and these flies show sensitivity to a range of extrinsic stressors. We show that expression of *Ndi1* partially rescues developmental arrest in mutants, and complex I deficiency-associated phenotypes in *dCIA30* RNAi knock down flies. These results support the idea that expression of *Ndi1* may be an effective therapeutic strategy to treat disorders resulting from defects in complex I assembly. Furthermore, our *dCIA30* knock down model provides a powerful tool to better understand the pathophysiological mechanisms of human disease arising from complex I deficiency.

## Materials and Methods

### *D. melanogaster* Culture

Flies were maintained on standard agar-cornmeal-yeast-sugar media [26] in humidified incubators at 25°C, on 12∶12 hour light:dark cycles. Flies were switched to new media every 2–3 days. Fly lines *CG7598^EY09101^* (stock #16925) [27] and *UAS-Gfp-IR* (stock #9331) were obtained from the Bloomington Drosophila Stock Center, and the *UAS-CG7598-IR* (stock #14859) fly line was obtained from the Vienna Drosophila RNAi Center [28]. *UAS-Ndi1* flies were generated as previously described [24]. An imprecise excision of the *CG7598^EY09101^* P element line, *dCIA30^ex80^*, was generated as previously described [29], and verified by sequencing. A precise excision that restores the gene was also generated, verified by sequencing, and used as controls in all experiments. *UAS-dCIA30* flies were generated by transforming flies with pUAST plasmids containing *dCIA30* cDNA using standard procedures.

### qRT-PCR

Total RNA was extracted using TRIzol reagent (Invitrogen, USA) following manufacturer protocols from 5 L3 larvae or 5 adult flies. Amplicons of *Actin5C* were used as a reference to normalize *dCIA30* amplicons. cDNA synthesis and qRT-PCR were performed in one step using Power SYBR Green RNA-to-CT 1-Step kit (Applied Biosystems, USA), and DNA amount was monitored during a 40-cycle PCR with an Applied Biosystems 7300 Thermal Cycler (Life Technologies, USA). Primer sequences: *Act5C*, TTGTCTGGGCAAGAGGATCAG and ACCACTCGCACTTGCACTTTC; *dCIA30*, TCACACCAAGGATGGCATTA and GCATGTTGTACTGCGTCCAG.

### Mitochondrial Isolation

Mitochondria were purified from larvae or adult flies by differential centrifugation as previously described [30]. Briefly, larvae or adults were homogenized in chilled mitochondrial isolation medium (MIM, 250 mM sucrose, 10 mM Tris (pH 7.4), 0.15 mM MgCl_2_) and debris was pelleted by centrifugation (500×g, 5 min at 4°C). Mitochondria were pelleted from the supernatant by centrifugation (5,000×g, 5 min at 4°C) and stored at −80°C.

### Blue-native Polyacrylamide Gel Electrophoresis (BN-PAGE)

BN-PAGE was performed using a Novex Native PAGE Bis-Tris Gel System (Invitrogen) following manufacturer protocols. Briefly, mitochondria were purified from 20 L3 larvae or 20 adult flies, resuspended in 25 µl of 1×Native PAGE Sample buffer (Invitrogen) with 1% digitonin and protease inhibitors (Roche, USA), and incubated on ice for 15 min. After centrifugation at 16,100×g for 30 min, 25 µl of the supernatant was resuspended with 1.25 µl of 5% G250 sample additive and 10 µl of 4×Native PAGE Sample Buffer (Invitrogen). Samples were loaded on 3–12% Bis-Tris Native PAGE gels and electrophoresed using 1×Native PAGE Running buffer system (Invitrogen). The cathode buffer included 1×Cathode Buffer Additive (Invitrogen). NativeMark Protein standard (Invitrogen) was used as the molecular weight marker. Protein concentrations of adult fly mitochondrial preps were determined with a Micro BCA Protein Assay Kit (Thermo Scientific, USA) following manufacturer instructions.

### Eclosion

Twenty wandering L3 larvae were collected in vials and maintained as described above. For approximately 10 days, the number of flies that eclosed from each vial was recorded daily.

### NADH:ubiquinone Oxidoreductase Activity Assay

Mitochondria were purified from 5 male L3 larvae, resuspended in 100 µl MIM, and 3 µl were added to 150 µl of a previously prepared colorimetric complex I activity assay buffer (1× PBS, 3.5 g/l BSA, 0.2 mM NADH, 0.24 mM KCN, 60 µM DCIP, 70 µM decylubiquinone, 25 mM antimycin A). NADH:ubiquinone oxidoreductase activity was monitored as a drop in DCIP absorbance at 600 nm using an Epoch microplate spectrophotometer (BioTek, USA). Flavone or rotenone insensitive activity was measured as the difference in DCIP reduction in the presence of flavone (0.4 mM) or rotenone (2 µM) in the assay buffer, and baseline activity in an assay buffer that contained both inhibitors. All reported activities are normalized to citrate synthase activity.

### Citrate Synthase Activity Assay

Mitochondrial preps used in the NADH:ubiquinone oxidoreductase assay were diluted 10 fold in MIM, and 3 µl were added to 150 µl of a previously prepared colorimetric citrate synthase activity assay buffer (50 mM Tris (pH 8.0), 0.1 mM 5,5′-dithiobis-(2-nitrobenzoic acid) (DTNB), 0.3 mM acetyl-CoA, 1 mM oxaloacetic acid). Citrate synthase activity was measured as an increase in DTNB absorbance at 412 nm using a microplate spectrophotometer.

### Electron Micrographs (EM)

EMs were acquired as previously described [29] from male flies 2 or 10 days post eclosion. Briefly, dorsal indirect flight muscle was dissected from decapitated adult flies at 4°C in 2% paraformaldehyde with 1% glutaraldehyde and fixed in the same solution overnight. After postfixation in 1% osmium tetroxide at room temperature, samples were dehydrated in an ethanol series and embedded in Epon 812. Ultrathin sections (80 nm) were examined with a Philips 420 electron microscope (Philips, Netherlands) at 100 kV at a magnification of 4900X.

### Weight

Flies were anesthetized under light N_2_ gas and weighed in groups of 5 in pre-weighed microcentrifuge tubes using an analytical scale (Torbal, USA).

### Stress Resistance

All stress assays were performed with male flies 6–8 days post eclosion in groups of 25–30 flies. For hypoxia resistance, flies were exposed to anoxic conditions in a 100% N_2_ chamber. Flies were maintained in the chamber for one hour, moved back into normoxia, and monitored for recovery (ability to stand) every 3 minutes for approximately 2 hours. For hyperoxia resistance, flies were maintained in a humidified chamber maintained at 85% O_2_ and survival was assayed at least once per day. For wet starvation, flies were maintained on water only medium (1% agar in ddH_2_O) and maintained in a 25°C incubator with 12 hour light:dark cycles. Survival was scored multiple times per day. For hyperthermia resistance, flies were maintained at 37°C and survival was scored every 2 hours.

### Statistical Analysis

Unless indicated otherwise, significance was determined with a two-tailed, unpaired *t* test from at least three independent experiments and expressed as p values. All error bars reflect standard error of the mean.

## Results

### *CG7598* (*dCIA30*), the *Drosophila* Homolog of Human Complex I Assembly Factor, *Ndufaf1*, is Necessary for Complex I Assembly

A *Drosophila melanogaster* homolog of the *Neurospora crassa* CIA30 protein, CG7598 (dCIA30), was previously identified in a homology search of amino acid sequences [31]. The dCIA30 protein shares high homology with human NDUFAF1 (69% similarity, 44% identity) in a ClustalW amino acid sequence alignment [32], in particular, in the C-terminal half where a conserved domain search turns up a conserved CIA30 domain (Figure S1A). As part of the *Drosophila* Gene Disruption Project [27], an insertion mutation of *dCIA30* (insertion EY09101, *dCIA30^EY09101^*) that contains an approximately 11 kb transposable P element (P{EPgy2}), in the 5′UTR is available and was acquired through the Bloomington stock center (stock #16925). In order to proceed with a line that disrupts the gene without the possible confounding presence of additional promoters, enhancers, and other genes, we generated additional lines in which the P{EPgy2} element was precisely and imprecisely excised by crossing *dCIA30^EY09101^* to flies harboring a transposase. One imprecise excision resulted in removal of the bulk of the P{EPgy2} element, leaving only a 517 bp fragment that does not carry any identifiable genetic elements, but does contain start and stop codons in all three forward frames (*dCIA30^ex80^*). In addition, a precise excision line was generated and used as a control throughout this study (Figure S1B).

**Figure 1:**
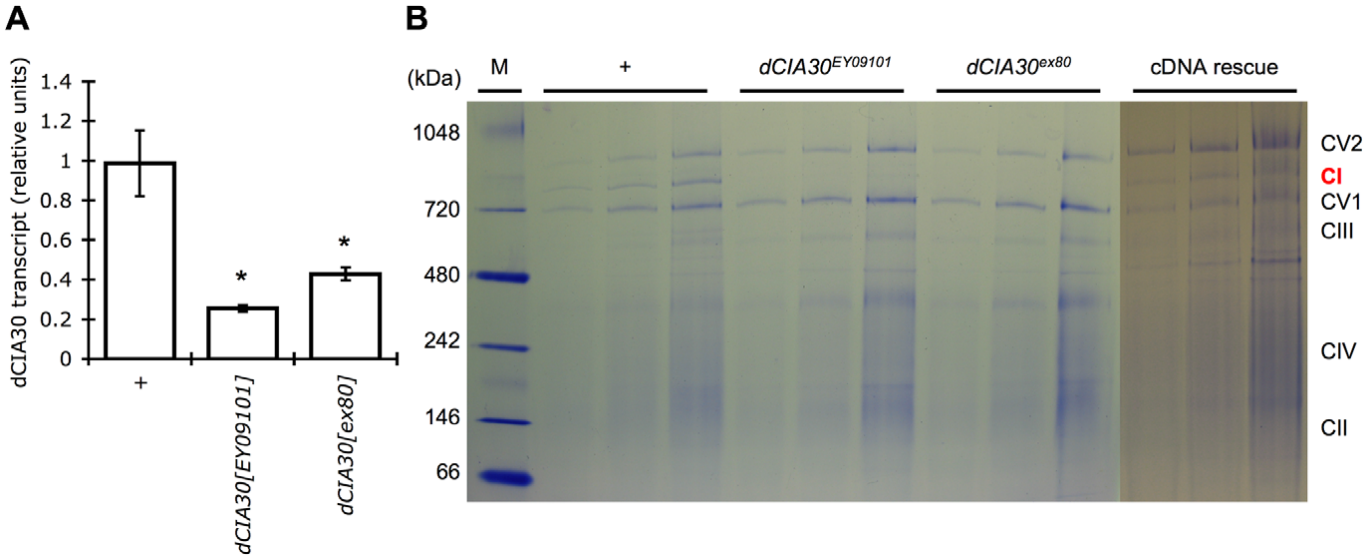
*dCIA30* mutation results in loss of complex I holoenzyme. (A) *dCIA30* transcript levels were measured by qRT-PCR in male third instar (L3) larvae (n = 5 male L3 larvae, 3 replicates). The *dCIA30^EY09101^* and *dCIA30^ex80^* insertion mutants show a *ca.* 55–75% loss of transcript relative to a precise excision control (* p<0.05). (B) The *dCIA30^EY09101^* and *dCIA30^ex80^* mutations result in a specific loss of the band that corresponds to complex I holoenzyme in blue native polyacrylamide gel electrophoresis (BN-PAGE) of L3 larvae. Precise excision controls (*+*) and larvae with expression of a *dCIA30* cDNA construct (*UAS-dCIA30/+; da-GAL4, dCIA30^ex80^/dCIA30^ex80^*, “cDNA rescue”) show the presence of the complex I holoenzyme band. (M = molecular size marker, CV2 = complex V dimer, CI = complex I, CV1 = complex V monomer, CIII = complex III, CIV = complex IV, CII = complex II, mitochondria from 2.5, 5, and 10 larvae equivalents in successive lanes for each genotype).

We checked the effects of the insertion mutation on *dCIA30* gene activity by quantifying *dCIA30* transcript levels during the third-instar larval stage of development (L3). Measurement of relative *dCIA30* mRNA levels by quantitative reverse transcriptase polymerase chain reaction (qRT-PCR) showed that both the *dCIA30^EY09101^* and *dCIA30^ex80^* homozygotes have a severe reduction of *dCIA30* mRNA levels relative to the control line (Figure 1A). We checked the effect of the reduced *dCIA30* mRNA levels on complex I assembly by BN-PAGE of mitochondria isolated from L3 larvae (Figure 1B). As expected, mitochondria from control larvae clearly show the presence of a band corresponding to complex I holoenzyme, identified by molecular mass. In contrast, the diminished levels of *dCIA30* in both the *dCIA30^EY09101^* and *dCIA30^ex80^* homozygotes are insufficient to support assembly and accumulation of complex I to detectable levels. Importantly, disruption of *dCIA30* did not noticeably affect the assembly or relative abundance of the other ETC complexes. To confirm that the complex I defect was due to loss of *dCIA30,* we expressed a wild-type *dCIA30* cDNA [33] via a ubiquitous *daughterless* promotor (*da-GAL4*), in a *dCIA30^ex80^* homozygous mutant background (*UAS-dCIA30/+; da-GAL4, dCIA30^ex80^/dCIA30^ex80^*). These ‘cDNA rescue’ flies clearly showed the presence of a complex I holoenzyme band (Figure 1B), demonstrating that the presence of exogenous dCIA30 is sufficient to rescue complex I assembly.

### Loss of dCIA30 Confers Developmental Arrest and Defects in Mitochondrial Function and Ultrastructure

**Figure 2:**
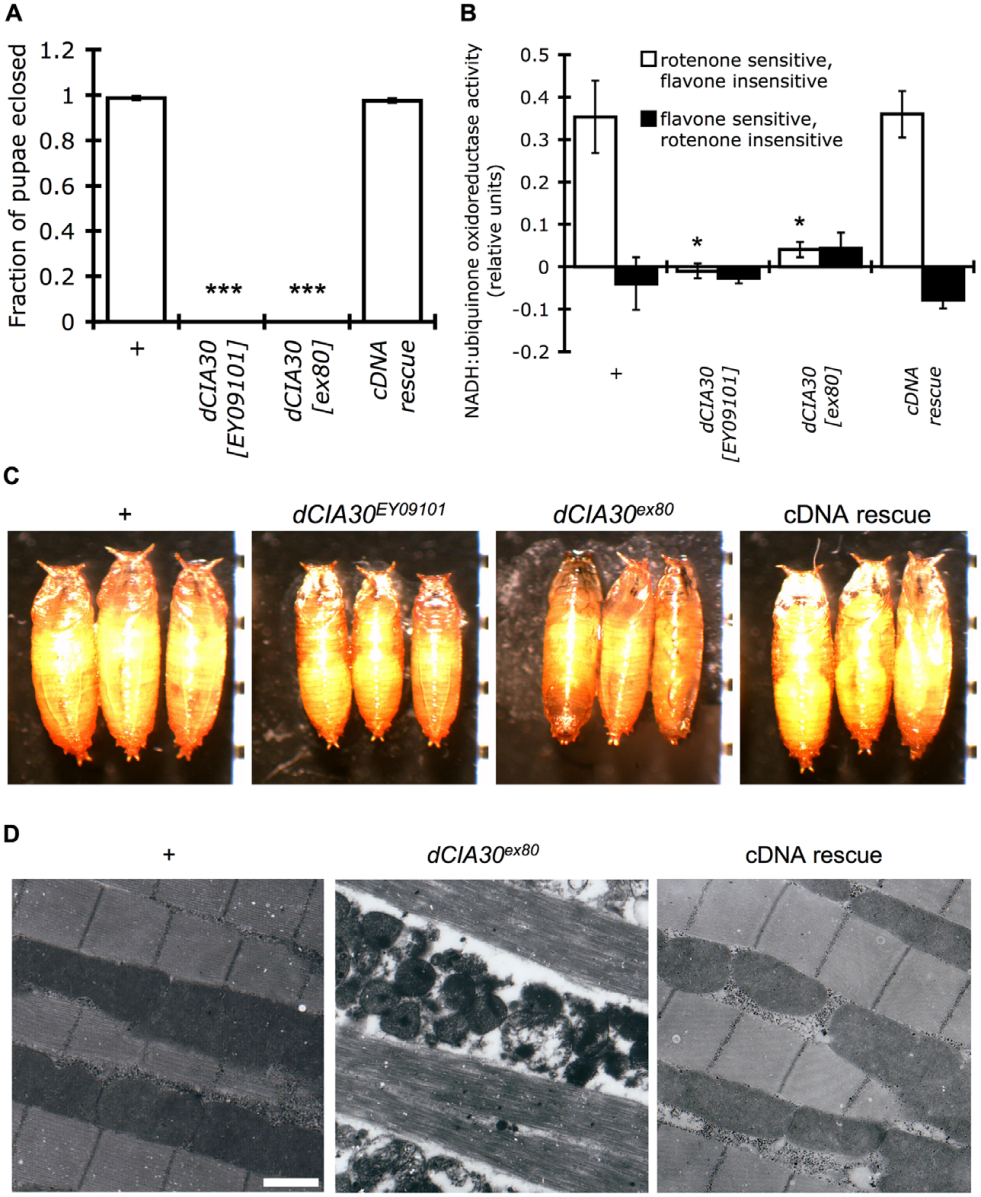
*dCIA30* mutation results in developmental arrest and defects in mitochondrial function and ultrastructure. (A) Homozygous *dCIA30^EY09101^* or *dCIA30^ex80^* mutants fail to develop past the pupal stage (***p<0.001). Expression of a cDNA rescue construct (cDNA rescue) is sufficient to rescue eclosion frequencies back to those of controls (*+*). (n = 20 L3 larvae, 8 replicates). (B) Isolated mitochondria from homozygous *dCIA30^EY09101^* or *dCIA30^ex80^* mutant male L3 larvae show negligible endogenous (rotenone sensitive, flavone insensitive) NADH:ubiquinone oxidoreductase activity relative to controls (+, * p<0.05). Expression of *dCIA30* cDNA restores endogenous NADH:ubiquinone oxidoreductase activity back to control levels. (n = 5 male L3 larvae, 5 replicates). (C) Homozygous *dCIA30^EY09101^* or *dCIA30^ex80^* pupae are structurally similar but visibly smaller relative to precise excision controls. Expression of *dCIA30* cDNA is sufficient to rescue the size deficit. Tick marks = 1 mm. (D) Electron micrographs (EMs) of flight muscle tissues show degeneration of myofibrils and interspersed mitochondria in homozygous *dCIA30^ex80^* male flies 2 days post eclosion. Expression of a *dCIA30* cDNA construct restores mitochondrial and myofibril integrity and organization. Scale bar = 1 µm.

Fly development proceeds through four distinct, easily identifiable stages, embryo, larva, pupa, and adult. Larvae homozygous for *dCIA30^EY09101^* or *dCIA30^ex80^* were viable and survived to late pupa stages. During late pupa/early adult stages, however, where approximately 100% of control pupae eclose to produce adults, a negligible fraction of pupae homozygous for *dCIA30^EY09101^* or *dCIA30^ex80^* developed into adult flies (Figure 2A). This developmental arrest was overcome by the transgenic expression of *dCIA30* in the cDNA rescue line. Both developmental arrest of mutant larvae and loss of complex I holoenzyme band correlated with loss of endogenous, rotenone sensitive, flavone insensitive NADH:ubiquinone oxidoreductase activity in isolated mitochondria from L3 larvae (Figure 2B). Pupae formed by the homozygous *dCIA30* mutants were visibly smaller than those formed by control and cDNA rescue larvae, but were structurally similar (Figure 2C).

In rare instances, adult *dCIA30^ex80^* homozygotes could be recovered by carefully assisting eclosing flies by manually peeling back the pupa case. Such escaper adults also showed an absence of a detectable complex I band in BN-PAGE analyses (Figure S2). In order to determine what effects *dCIA30* mutation and loss of complex I holoenzyme had on mitochondrial ultastructure, we examined electron micrographs (EMs) of thoracic sections of precise excision control flies, homozygous *dCIA30^ex80^* escaper flies, and cDNA rescue flies (Figure 2D). The thoracic flight muscle tissue of adult flies is highly organized and consists of myofibrils interspersed with densely packed mitochondria. Flight muscle tissue from control flies showed highly ordered and intact myofibrils and mitochondria, whereas homozygous *dCIA30^ex80^* escaper flies showed severe degeneration of both myofibrils and mitochondria. Both mitochondrial and myofibril phenotypes were rescued in the cDNA rescue flies.

### RNAi of *dCIA30* Leads to Complex I Holoenzyme Loss

**Figure 3:**
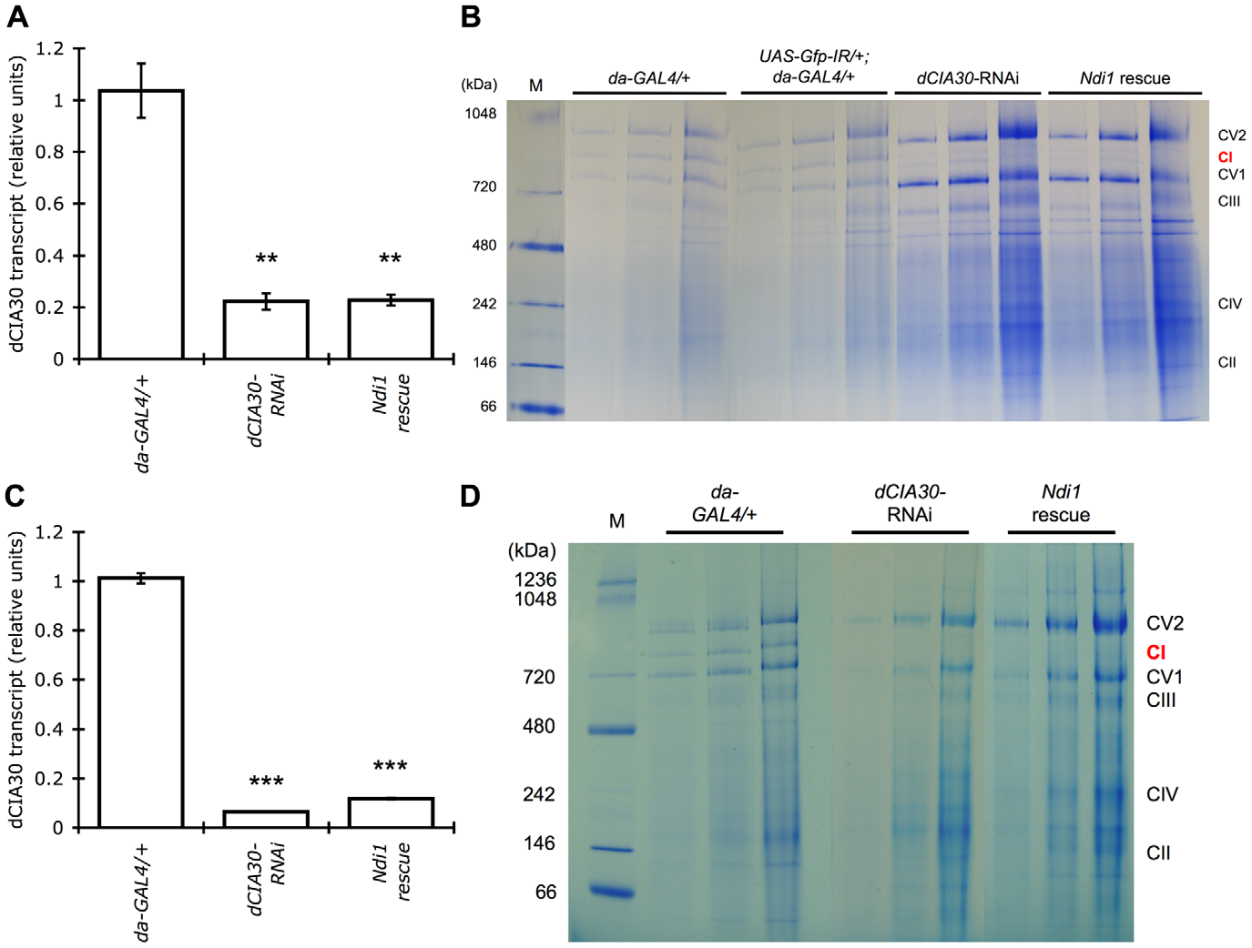
RNAi knock down of *dCIA30* confers loss of complex I holoenzyme. (A) Expression of an RNAi mediated knockdown construct specific for *dCIA30* (*da-GAL4/UAS-dCIA30-IR*) reduces *dCIA30* transcript levels (*dCIA30-*RNAi, **p<0.01) in L3 larvae relative to controls (*da-GAL4/+*). Co-expression of a *UAS-Ndi1* transgene (*da-GAL4/UAS-dCIA30-IR, UAS-Ndi1*, “*Ndi1* rescue”) with the same driver has little effect on the *dCIA30* knock down. (n = 5 L3 larvae, 3 replicates). (B) RNAi knock down of *dCIA30* results in a dramatic reduction of the band that corresponds to complex I holoenzyme in BN-PAGE of L3 larvae. In comparison, driver only controls (*da-GAL4/+*) and RNAi controls (*UAS-Gfp-IR/+; da-GAL4/+*) show the presence of a distinct complex I holoenzyme band. Co-expression of a *UAS-Ndi1* transgene in the *Ndi1* rescue line does not affect the reduction of the complex I holoenzyme band. (M = molecular size marker, CV2 = complex V dimer, CI = complex I, CV1 = complex V monomer, CIII = complex III, CIV = complex IV, CII = complex II, mitochondria from 2.5, 5, and 10 larvae equivalents in successive lanes for each genotype). (C) *dCIA30* transcript levels are reduced in male *dCIA30-*RNAi flies, 6 days post eclosion. Expression of the *UAS-dCIA30-IR* knockdown construct reduces *dCIA30* transcript levels (***p<0.001) to approximately 6% relative to control. Co-expression of *UAS-Ndi1* (*Ndi1* rescue) has no major effect on *dCIA30* transcript levels. (n = 5 male flies, 3 replicates). (D) RNAi knock down of *dCIA30* results in reduction of the band that corresponds to complex I holoenzyme to undetectable levels in BN-PAGE of male flies 2 days post eclosion. In comparison, RNAi controls show the presence of a distinct complex I holoenzyme band. Co-expression of a *UAS-Ndi1* transgene does not affect the loss of the complex I holoenzyme band. (10, 25, 50 µg of total mitochondrial protein in successive lanes for each genotype).

The developmental arrest of *dCIA30* mutants was a confounding factor in characterizing complex I loss in adult flies. RNAi knock down of *dCIA30* (*da-GAL4/UAS-dCIA30-IR*, referred to as “*dCIA30*-RNAi” in this report) provided a means to phenocopy a less severe *dCIA30* mutation. A UAS-hairpin RNAi knockdown construct targeted to the first exon (Figure S1B) that has been reported to have no off-target effects was acquired from the Vienna Drosophila RNAi Center (stock #14859) [28]. Expression of the knockdown construct using *da-GAL4* resulted in a significant reduction of *dCIA30* mRNA levels during the L3 stage of development (Figure 3A). Accordingly, there was a dramatic reduction in the band corresponding to complex I holoenzyme in BN-PAGE analyses in *dCIA30* RNAi knockdown larvae (Figure 3B). Unlike the *dCIA30* mutant larvae, however, these larvae still had a faint but detectable complex I holoenzyme band. A *Gfp*-RNAi line did not impact complex I levels, indicating that the loss of complex I was not due to non-specific effects of RNAi. Adult flies also had reduced expression of *dCIA30* (Figure 3C) and no detectable complex I holoenzyme (Figure 3D).

**Figure 4:**
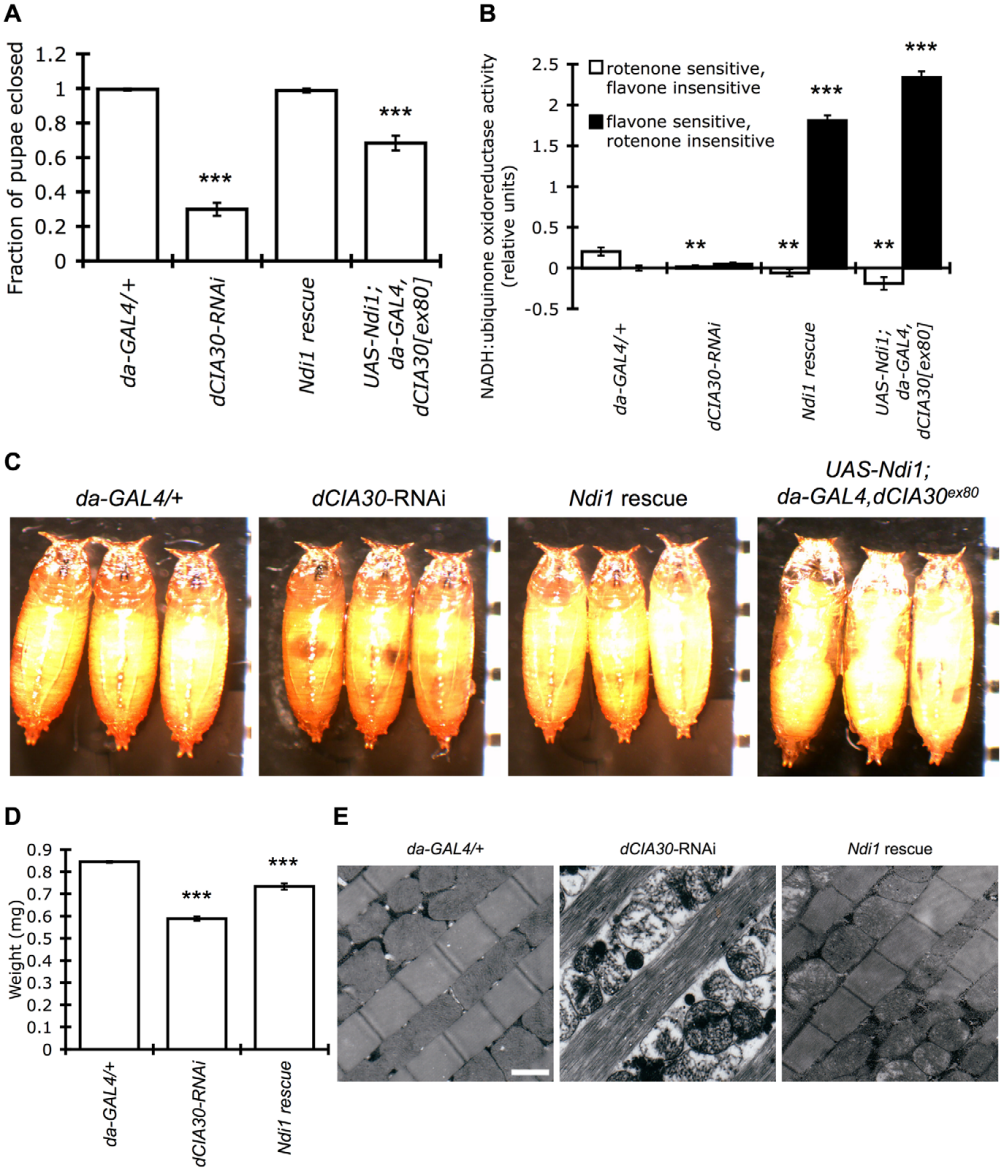
*dCIA30* knockdown flies display developmental and physiological phenotypes that are rescued by NDI1. (A) RNAi knock down of *dCIA30* (*dCIA30-*RNAi) results in a significant drop in the frequency of pupae that eclose to adult flies relative to controls (*da-GAL4/+*, ***p<0.001). Co-expression of NDI1 in *dCIA30*-RNAi flies (*Ndi1* rescue) restores eclosion back to levels found in control pupae. The expression of NDI1 from double copies of both *UAS-Ndi1* and *da-GAL4* in *dCIA30^ex80^* insertion mutants (*UAS-Ndi1;da-GAL4,dCIA30^ex80^*) restores eclosion to approximately 70% of pupae (***p<0.001). (n = 20 L3 larvae, 8 replicates). (B) Mitochondria isolated from male L3 larvae with RNAi knock down of *dCIA30* (*dCIA30*-RNAi) show negligible endogenous (rotenone sensitive, flavone insensitive) NADH:ubiquinone oxidoreductase activity relative to controls (*da-GAL4/+*), regardless of *UAS-Ndi1* co-expression (*Ndi1* rescue, **p<0.01). Expression of *UAS-Ndi1* causes a dramatic increase in flavone sensitive, rotenone insensitive NADH:ubiquinone oxidoreductase activity in a *dCIA30* knock down (*Ndi1* rescue) or mutant background (*UAS-Ndi1;da-GAL4,dCIA30^ex80^*, ***p<0.001). (n = 5 male L3 larvae, 5 replicates). (C) RNAi knock down of *dCIA30* produces pupae that are smaller relative to controls. The expression of *UAS-Ndi1* does not result in rescue of the pupal phenotype in RNAi knock down flies. However, expression of NDI1 with double copies of both transgene and driver (*UAS-Ndi1;da-GAL4,dCIA30^ex80^*) increases pupal size in *dCIA30^ex80^* mutants (See figure 2C for comparison). Tick marks = 1 mm. (D) Adult male *dCIA30* knock down flies, 6 days post eclosion have significantly lower body weights compared to controls (***p<0.001). Co-expression of NDI1 in these knock down flies partially restores body weight (***p<0.001). (n = 5 male flies, 6 replicates). (E) EMs of thoracic flight muscles from male *dCIA30* knock down flies show degeneration of mitochondria and myofibrils at 10 days post eclosion. Co-expression of NDI1 largely restores mitochondrial and myofibril integrity and organization. Scale bar = 1 µm.

Unlike homozygous mutation of *dCIA30*, which caused an essentially complete arrest of development at pupation, RNAi knockdown of *dCIA30* retained some complex I holoenzyme during larval stages. One physiological consequence of the milder knockdown was the eclosion of adult flies in reduced, but significant numbers (Figure 4A). As was the case for *dCIA30* mutants, RNAi knock down of *dCIA30* resulted in small pupae (Figure 4C).

### Yeast *Ndi1* can Partially or Wholly Rescue Phenotypes Associated with *dCIA30* Deficiency

Next, we set out to determine whether the alternative NADH dehydrogenase, *Ndi1*, could complement the loss of *dCIA30*. Expression of NDI1 (*da-GAL4/UAS-dCIA30-IR,UAS-Ndi1*, referred to as “*Ndi1* rescue” in this report) in flies with RNAi knock down of *dCIA30* completely reverted the developmental arrest phenotype to control levels (Figure 4A) and expression of NDI1 from two copies each of *UAS-Ndi1* and *da-GAL4* transgenes (*UAS-Ndi1*;*da-GAL4*,*dCIA30^ex80^*) resulted in a significant rescue of the mutant phenotypes (Figure 4A–C). Adult males that eclosed from these pupae were able to fertilize wild type females whereas adult females failed to produce offspring in crosses with wild type male flies.

This rescue was not a result of a cryptic increase in complex I assembly by NDI1 or by dilution of the *UAS-GAL4* system. NADH:ubiquinone oxidoreductase activity of isolated mitochondria from L3 larvae showed negligible endogenous rotenone sensitive, flavone insensitive activity in the knock down lines, relative to controls, even in the presence of *UAS-Ndi1* transgenes (Figure 4B). Instead, expression from *UAS-Ndi1* transgenes resulted in large increases in flavone sensitive, rotenone insensitive activity that corresponds to NDI1 activity (Figure 4B). Moreover, the expression of two copies of *UAS-Ndi1* construct in a homozygous *dCIA30^ex80^* background (*UAS-Ndi1;da-GAL4,dCIA30^ex80^*), did not show any effect on the missing complex I band (Figure S3). Similarly, the co-expression of a *UAS-Ndi1* transgene in *dCIA30* RNAi knockdown lines using the same *UAS-GAL4* system had little effect on *dCIA30* mRNA knockdown (Figure 3A and 3C) or in the resulting defect in complex I holoenzyme assembly (Figure 3B and 3D).

RNAi knockdown of *dCIA30* led to an approximately 30% reduction in body weight relative to driver only controls (*da-GAL4/+*). Co-expression of NDI1 largely restored the adult body weight of flies with *dCIA30* knockdown (Figure 4D). RNAi of *dCIA30* also resulted in detrimental effects on mitochondrial and myofibril structure in adult flies. EM analysis of thoracic muscle revealed severe degeneration of mitochondria and myofibrils, similar to those seen in *dCIA30* mutant flies (Figure 4E). Co-expression of NDI1 was sufficient to fully rescue the myofibril defect and partially rescue the mitochondrial ultrastructure defect.

**Figure 5:**
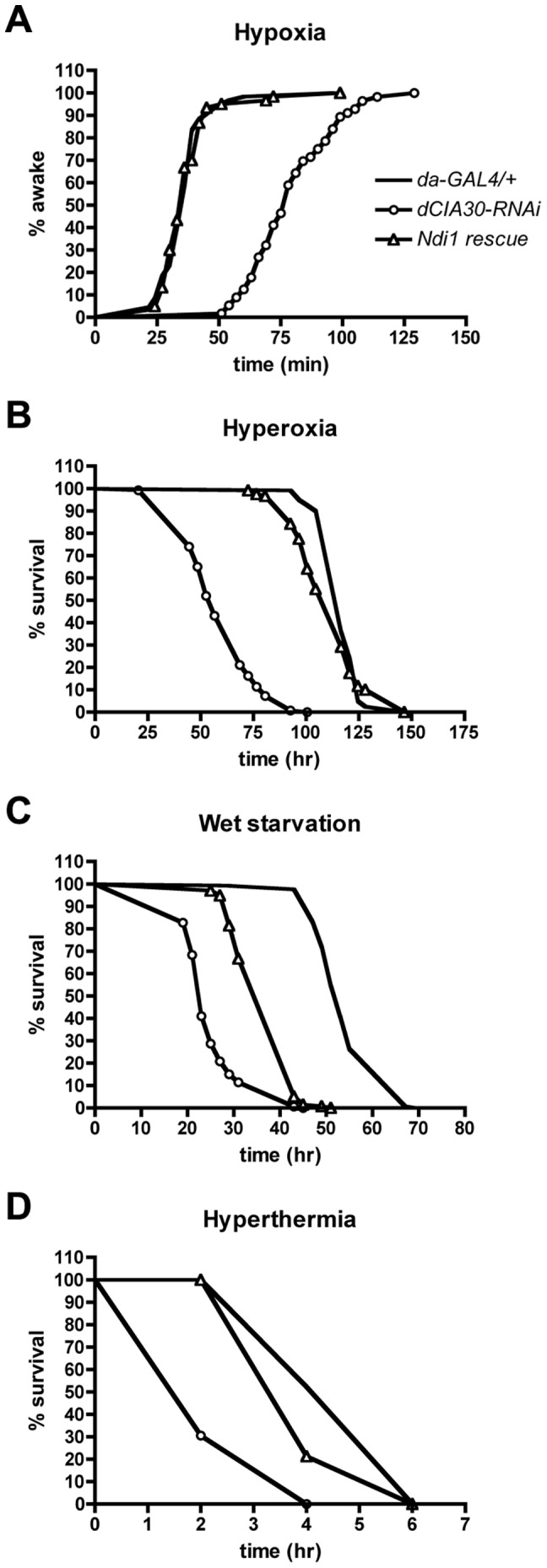
Loss of *dCIA30* results in multiple stress sensitivity phenotypes that are ameliorated by NDI1. (A) Knock-down of *dCIA30* (*dCIA30-*RNAi) in adult flies increased recovery time after exposure to hypoxia (1 hr). Loss of *dCIA30* more than doubled mean recovery time compared to controls (*da-GAL4/+*). Co-expression of NDI1 (*Ndi1* rescue) restored recovery time to control levels. (n = 120 male flies). (B) Knock down of *dCIA30* in adult flies decreased survival under hyperoxia (84% O_2_). On average, *dCIA30-*RNAi flies lived approximately half as long as controls. Co-expression of NDI1 restored mean survival duration to approximately 95% of controls. (n = 120 male flies). (C) Knock down of *dCIA30* in adult flies decreased survival duration under starvation with water. On average, flies with dCIA30 knock down survived less than half as long as control flies. Co-expression of NDI1 increased survival times to approximately 70% of controls. (n>120 male flies). (D) Knock down of *dCIA30* in adult flies decreased survival duration under high temperature (37°C). Mean survival times of flies with reduced *dCIA30* were approximately half those of control flies. Co-expression of NDI1 increased mean survival time to approximately 90% of controls. (n>135 male flies).

To quantify the physiological effects of complex I loss, we tested the ability of *dCIA30* knockdown flies to cope with various forms of extrinsic stress. To explore the role of complex I/NDI1 in the ability to withstand oxidative stress, we examined recovery from hypoxia and survivorship under hyperoxia. RNAi knock down of *dCIA30* conferred a drastically reduced ability to recover from exposure to hypoxia, which was completely rescued by NDI1 expression (Figure 5A). Flies with reduced expression of *dCIA30* also showed increased sensitivity to hyperoxia, which was also rescued by NDI1 expression (Figure 5B). Loss of complex I is expected to have drastic effects on metabolism, and accordingly, flies without detectable complex I were much more susceptible to wet starvation (Figure 5C). Providing an alternative source of mitochondrial NADH dehydrogenase activity by expressing NDI1 partially restored survival under starvation conditions. Similarly, loss of complex I drastically reduced resistance to elevated temperature that was largely restored by NDI1 expression (Figure 5D).

## Discussion

In the current report, we have demonstrated the role of *CG7598/dCIA30*, the *Drosophila* homolog of human *Ndufaf1*, as a vital factor in the assembly of mitochondrial complex I. We show that loss of *dCIA30* is sufficient to reduce complex I holoenzyme to levels that are undetectable in BN-PAGE. Reduction of complex I holoenzyme levels can be reverted by precise excision of the inserted element in the *dCIA30* mutants or by expression of a wild type *dCIA30* cDNA construct. These findings confirm that *dCIA30* is a necessary factor for complex I assembly in *Drosophila*. *dCIA30* mutation almost completely abrogated development during the pupa stage, and reduced complex I holoenzyme levels in *dCIA30* RNAi knockdown flies caused an approximately 70% drop in eclosion frequency. In flies that did reach adulthood, *dCIA30* knock down further reduced complex I holoenzyme to undetectable levels in BN-PAGE. In addition to being smaller, these flies showed dramatically increased sensitivity to a variety of extrinsic stressors including hypoxia, hyperoxia, hyperthermia, and starvation. Interestingly, while sensitivity to hypoxia, hyperoxia and high temperature (conferred by loss of complex I) can be completely or almost completely rescued by *Ndi1*, sensitivity to starvation conditions was only partially rescued by *Ndi1*. Therefore, transkingdom gene therapy using *Ndi1* may have limitations with respect to complementing defects of energy metabolism.

Among other detriments, loss of complex I may result in reduced ETC function, increased oxidative stress, skewing of the NAD:NADH ratio, and reduced ability to form supercomplexes [7]. Of these, loss of ETC activity, increased oxidative stress, and skewing of the NAD:NADH ratio may be improved by supplementation with NDI1 [18], [24], [34]. In some fungi, such as *S. cerevisiae*, NDI1 functions as the sole matrix facing NADH:ubiquinone oxidoreductase [15], coupling NADH oxidation to ubiquinone reduction like mitochondrial complex I, but without translocating protons across the inner mitochondrial membrane. As *Ndi1* in the present study was cloned from *S. cerevisiae* [24], it is unlikely that it could act as a scaffold for supercomplex formation or otherwise participate specifically and directly in other endogenous pathways when expressed in *Drosophila*, which does not have an endogenous *Ndi1* homolog. The near complete rescue of sensitivity to hypoxia, hyperoxia, and hyperthermia in complex I deficient flies by NDI1 supplementation suggests that sensitivity to hypoxia, hyperoxia, and hyperthermia may be linked to loss of ETC activity, increased oxidative stress, and/or skewing of the NAD:NADH ratio. Conversely, the major detriment in wet starvation may be a result of a complex I function that is not supplemented or insufficiently supplemented by NDI1 function. Although beyond the scope of this work, a further analysis of the contribution of the different harmful effects of complex I deficiency to various stresses may yield further insights into the etiology of complex I deficiencies.

NDI1 has previously been shown to be effective as a therapeutic for complex I disorders, including fly [18], mouse [19], and rat [20] models of Parkinson’s disease, and in the fungus *Podospora anserina*, overexpression of endogenous *Ndi1* was shown to be able to rescue complex I holoenzyme deficiency [14]. To our knowledge, however, this is the first demonstration that exogenous NDI1 is able to restore development to a metazoan that has no detectable complex I holoenzyme in BN-PAGE or NADH:ubiquinone oxidoreductase activity in a colorimetric assay. Moreover, the generation and characterization of a fly model of complex I assembly factor deficiency will facilitate future work into the underlying pathophysiology of neurodegeneration and aging.

## Supporting Information

Figure S1 CG7598 is the D. melanogaster homolog of Ndufaf1/CIA30. (A) The *D. melanogaster* homolog of human *Ndufaf1*, *CG7598* (*dCIA30*) was identified by homology. Amino acid sequence alignment shows 69% similarity (44% identity), concentrated near the C-terminal half, which contains the CIA30 domain. (B) *CG7598* (*dCIA30*) maps to chromosome 3R at 99B9 and spans approximately 1.1 kb. The coding sequence consists of two exons separated by a 58 bp intron. A fly line with an insertion of an approximately 11 kb long P-element (P{EPgy2}) into the 5′UTR, 96 bp downstream from the transcriptional start site (*dCIA30^EY09101^*) was used for initial mutant studies. A line that contains a smaller insertion of approximately 500 bp was generated by imprecise excision of the transposable element (*dCIA30^ex80^*). A hairpin RNAi construct targeting the 325 bp of the first exon with no reported off target knock downs (*UAS-dCIA30-IR*) was used for RNAi knock down studies.
(TIF)

Figure S2 Complex I loss due to *dCIA30* mutation persists in adult flies. The *dCIA30^ex80^* mutation results in specific loss of complex I holoenzyme band in BN-PAGE of adult male flies, 2 days post eclosion. In contrast, controls (*+*) and cDNA rescue flies show the presence of the complex I holoenzyme band. (M = molecular size marker, CV2 = complex V dimer, CI = complex I, CV1 = complex V monomer, CIII = complex III, CIV = complex IV, CII = complex II, 10, 25, 50 µg of total mitochondrial protein in successive lanes for each genotype).
(TIF)

Figure S3 Expression of NDI1 does not affect complex I assembly. Expression of a *UAS-Ndi1* construct does not affect the absence of the complex I holoenzyme band in a *dCIA30^ex80^* mutant background. (M = molecular size marker, CV2 = complex V dimer, CI = complex I, CV1 = complex V monomer, CIII = complex III, CIV = complex IV, CII = complex II, mitochondria from 2.5, 5, and 10 larvae equivalents in successive lanes).
(TIF)
