# Supplementary figures and images for: Expression of Yeast NDI1 Rescues a Drosophila Complex I Assembly Defect
Source: PLoS One. 2012 Nov 30;7(11):e50644. doi: 10.1371/journal.pone.0050644 (PMC3511326; doi:10.1371/journal.pone.0050644)

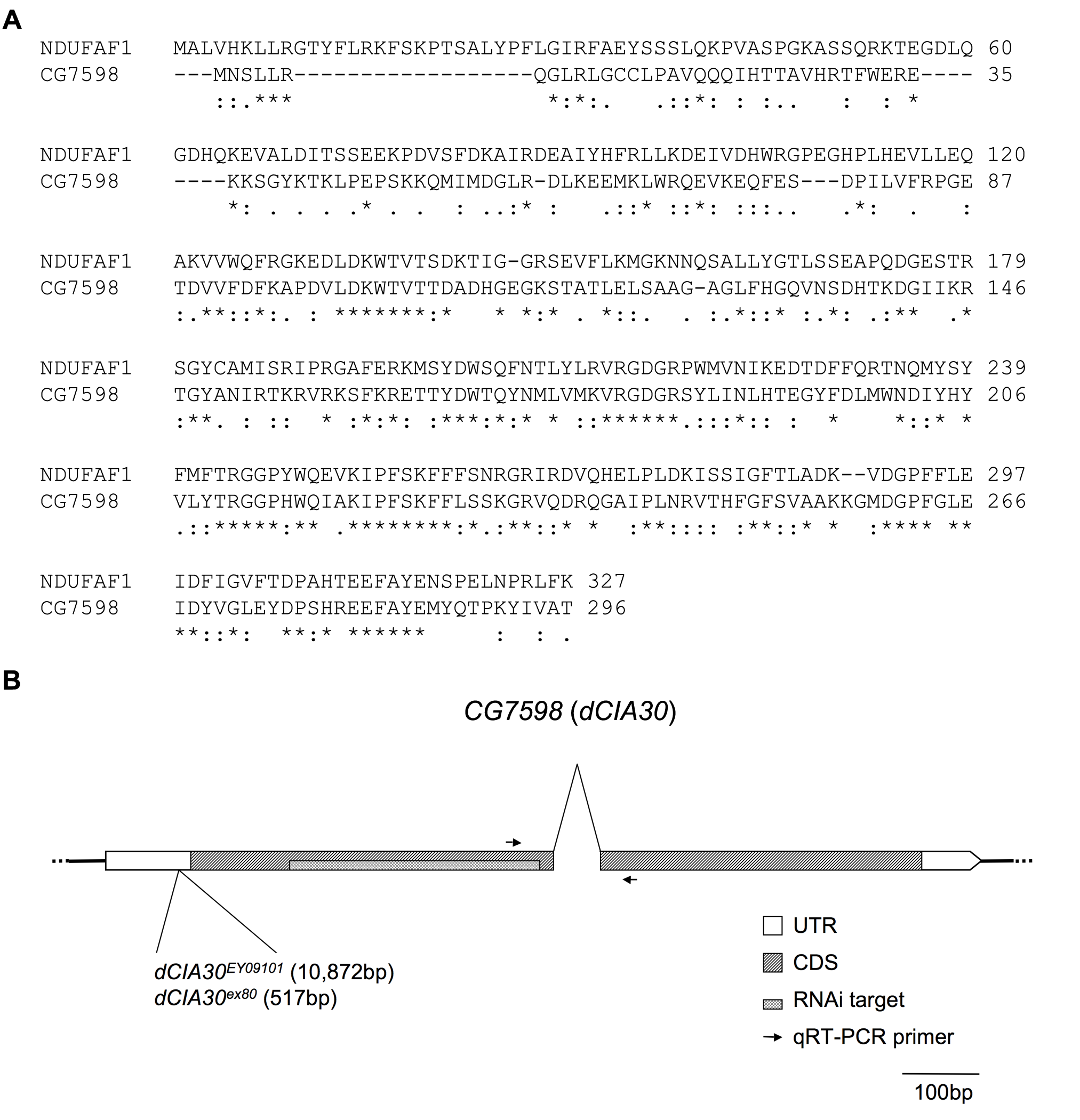

Supplement: Figure S1 — CG7598 is the D. melanogaster homolog of Ndufaf1/CIA30. (A) The D. melanogaster homolog of human Ndufaf1, CG7598 (dCIA30) was identified by homology. Amino acid sequence alignment shows 69% similarity (44% identity), concentrated near the C-terminal half, which contains the CIA30 domain. (B) CG7598 (dCIA30) maps to chromosome 3R at 99B9 and spans approximately 1.1 kb. The coding sequence consists of two exons separated by a 58 bp intron. A fly line with an insertion of an approximately 11 kb long P-element (P{EPgy2}) into the 5′UTR, 96 bp downstream from the transcriptional start site (dCIA30EY09101) was used for initial mutant studies. A line that contains a smaller insertion of approximately 500 bp was generated by imprecise excision of the transposable element (dCIA30ex80). A hairpin RNAi construct targeting the 325 bp of the first exon with no reported off target knock downs (UAS-dCIA30-IR) was used for RNAi knock down studies. (TIF) [file pone.0050644.s001.tif]

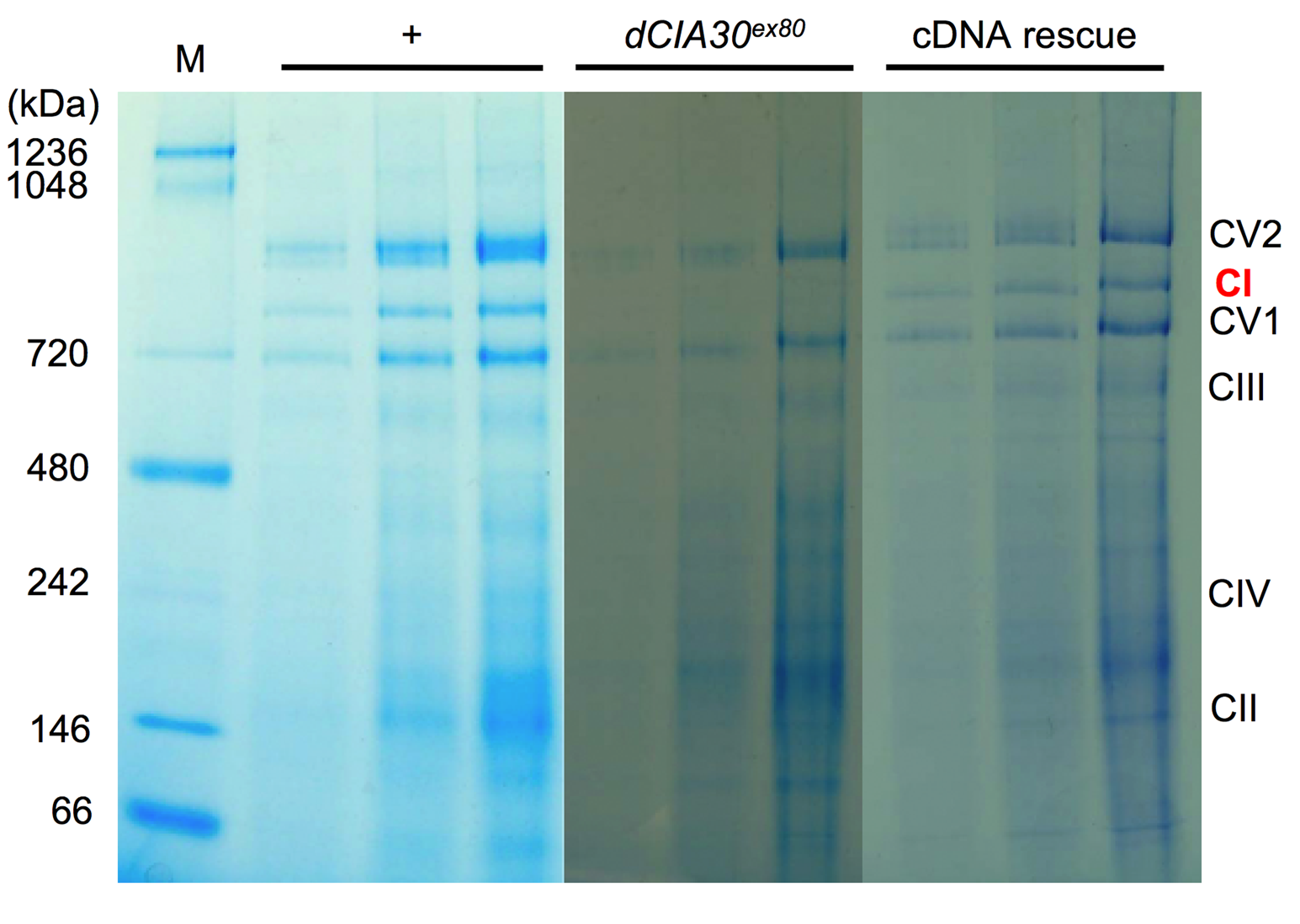

Supplement: Figure S2 — Complex I loss due to dCIA30 mutation persists in adult flies. The dCIA30ex80 mutation results in specific loss of complex I holoenzyme band in BN-PAGE of adult male flies, 2 days post eclosion. In contrast, controls (+) and cDNA rescue flies show the presence of the complex I holoenzyme band. (M = molecular size marker, CV2 = complex V dimer, CI = complex I, CV1 = complex V monomer, CIII = complex III, CIV = complex IV, CII = complex II, 10, 25, 50 µg of total mitochondrial protein in successive lanes for each genotype). (TIF) [file pone.0050644.s002.tif]

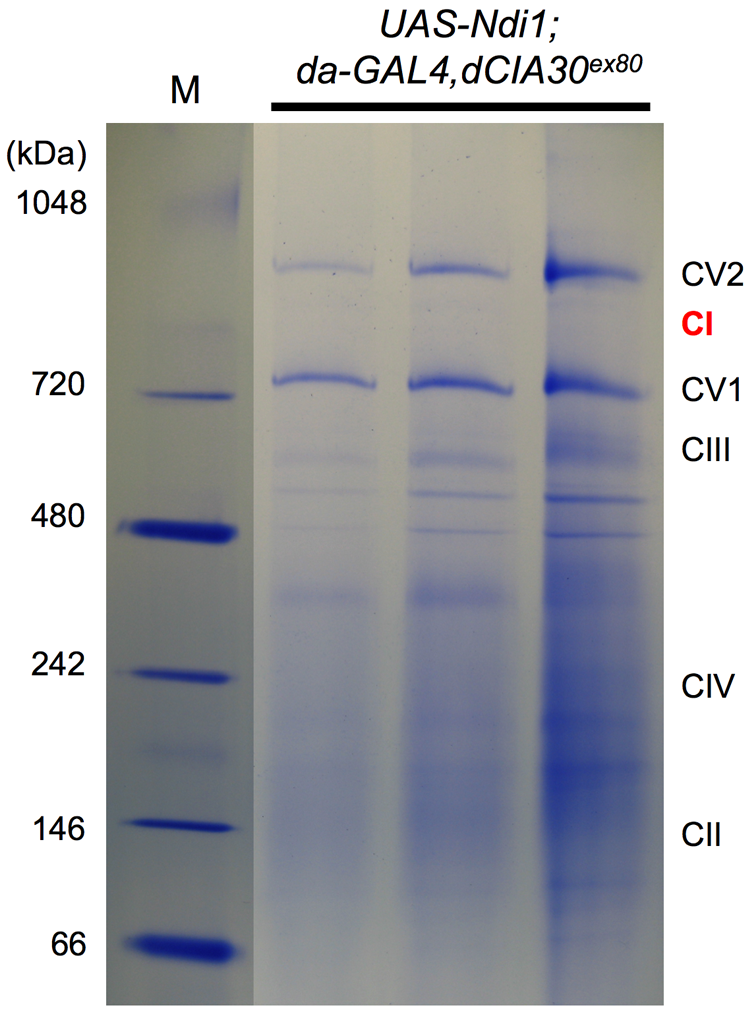

Supplement: Figure S3 — Expression of NDI1 does not affect complex I assembly. Expression of a UAS-Ndi1 construct does not affect the absence of the complex I holoenzyme band in a dCIA30ex80 mutant background. (M = molecular size marker, CV2 = complex V dimer, CI = complex I, CV1 = complex V monomer, CIII = complex III, CIV = complex IV, CII = complex II, mitochondria from 2.5, 5, and 10 larvae equivalents in successive lanes). (TIF) [file pone.0050644.s003.tif]
